# Sacrococcygeal teratoma: Excision aided by laparocopic ligation of the median sacral artery in a premature neonate

**DOI:** 10.4103/0971-9261.54807

**Published:** 2009

**Authors:** A. P. Desai, R. Wragg, M. Kulkarni, T. Tsang

**Affiliations:** Department of Pediatric Surgery, Norfolk & Norwich University Hospital, Colney Lane, Norwich NR4 7UY, UK

Sir,

Sacrococcygeal teratoma (SCT) is the most common teratoma presenting at birth. It can have variable intrapelvic extension. One of the most immediate risks with sacrococcygeal teratoma is the risk of life threatening hemorrhage, either perinatally or during surgical resection.

In the presence of significant intrapelvic extension, ligation of the median sacral artery during laparotomy has been used to minimise this risk during surgery.[1] Recently, there have been a few reports of the use of laparoscopic ligation of the artery.[2–4] We report the successful use of this technique in a premature newborn infant.

The diagnosis of sacrococcygeal teratoma had been made antenatally at 22 weeks gestation. The parents were counselled. The mother went into pre- mature labor, and the female infant was delivered by emergency caesarean section at 30+2 weeks. The birth weight was 1.826 kg. She required ventilation for 12 minutes at birth and was then commenced on continuos positive pressure airway ventillation.

The external component of the teratoma measured 5.7cm in diameter. An ultrasound scan showed that the internal component filled the entire true pelvis with an intra-pelvic extension of 6 cm. It was related to the sacral promontory and displaced the rectum and bladder superiorly. It was mainly solid with some cystic components and foci of calcification. Hence, diagnosis of Type 3 SCT was made according to Altman's classification.

At the beginning of the surgery, the patient was initially placed supine. A laparoscopy surgery was performed using a 5mm, 0 degree laparoscope. The laparoscope was placed at the umbilicus using Hassan's technique. Another 5 mm port was placed in the right iliac fossa and a 3 mm port was placed in the left iliac fossa. The retroperitoneal space was identified next to the rectum and opened by a sharp and blunt dissection. A single median sacral artery was identified and clipped in continuity using two 5 mm endoclips. The patient was then turned to a prone position and a cruciate, perineal incision was performed. The teratoma was dissected from the surrounding muscles and connective tissue. The tumor was excised en bloc with the tip of the coccyx in a standard surgical technique.

Total estimated blood loss was 15 ml. The dimensions of the teratoma were 9 cm × 5 cm × 7 cm and weighed 78 grams. A penrose drain was left *in situ* but drained minimally and was removed 48 hours later. Postoperatively the patient remained well and was extubated the same day. She was kept in the neonatal intensive care unit in view of her premature status and was discharged later.

The hemorrhage of a sacrococcygeal teratoma is a life-threatening event and the proper care must be taken to avoid it. The ligation of the median sacral artery has long been a useful technique in controlling the blood supply of these tumors, thus reducing the risk of hemorrhage.[1]

For tumors with significant intrapelvic extension, ligation can be performed by a laparotomy. Laparoscopic ligation of the median sacral artery is an alternative method of controlling the risk, which does not require a laparotomy. This has been reported previously in literature as early as 1998 as a safe and effective procedure.[2–4]

This report shows that the use of this technique is both possible and safe in a premature infant with low birth weight. Our patient is the smallest infant so far reported where this technique has been used.
